# Interaction of Imidazole Containing Hydroxamic Acids with Fe(III): Hydroxamate Versus Imidazole Coordination of the Ligands

**DOI:** 10.1155/2007/96536

**Published:** 2007-12-30

**Authors:** Etelka Farkas, Dávid Bátka, Hajnalka Csóka, Nóra V. Nagy

**Affiliations:** ^1^Department of Inorganic and Analytical Chemistry, University of Debrecen, 4010 Debrecen, Hungary; ^2^Central Research Center, Hungarian Academy of Sciences, 1525 Budapest, Hungary

## Abstract

Solution equilibrium studies on Fe(III) complexes formed with imidazole-4-carbohydroxamic acid (Im-4-Cha), N-Me-imidazole-4-carbohydroxamic acid (N-Me-Im-4-Cha), imidazole-4-acetohydroxamic acid (Im-4-Aha), and histidinehydroxamic acid (Hisha) have been performed by using pH-potentiometry, UV-visible spectrophotometry, EPR, ESI-MS, and H1-NMR methods. All of the obtained results demonstrate that the imidazole moiety is able to play an important role very often in the interaction with Fe(III), even if this metal ion prefers the hydroxamate chelates very much. If the imidazole moiety is in α-position to the hydroxamic one (Im-4-Cha and N-Me-Im-4-Cha) its coordination to the metal ion is indicated unambiguously by our results. Interestingly, parallel formation of (${\text{N}}_{\text{imidazole}}$, ${\text{O}}_{\text{hydroxamate}}$), and (${\text{O}}_{\text{hydroxamate}}$, ${\text{O}}_{\text{hydroxamate}}$) type chelates seems probable with N-Me-Im-4-Cha. The imidazole is in β-position to the hydroxamic moiety in Im-4-Aha and an intermolecular noncovalent (mainly H-bonding) interaction seems to organize the intermediate-protonated molecules in this system. Following the formation of mono- and bishydroxamato mononuclear complexes, only EPR silent species exists in the Fe(III)-Hisha system above pH 4, what suggests the rather significant “assembler activity” of the imidazole (perhaps together with the ammonium moiety).

## 1. INTRODUCTION

**Scheme 1:**
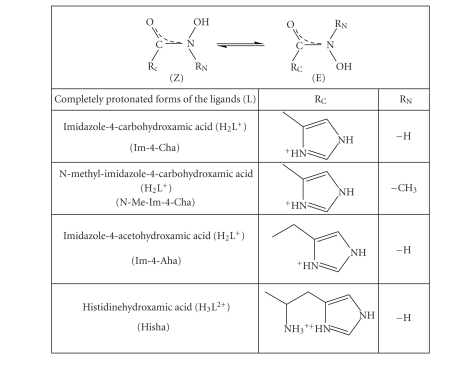
Formulae of the studied ligands.

The best-known hydroxamic acids belong to the microbial siderophores [1]. These molecules quite generally contain three chelating functions and are responsible for selective uptake and transport of Fe(III) into the cells in adequate concentration. Because of the long-term usage of desferrioxamine B (DFB), first of all for the treatment of thalassemic patients, perhaps this compound is the most famous representative of the hydroxamate-based siderophore family [2, 3]. Not only natural, but also synthetic hydroxamic acids are effective metal binding agents and their numerous biological effects (e.g., capability for inhibition of various metalloenzymes) are strongly connected to their metal complexation [4, 5]. Although their Fe(III) complexes are the most stable nearly in all cases, they are also capable of strong interaction with numerous other metals [6]. Moreover, the preference of Fe(III) over some 3d M(II) metals, like Cu(II) or Ni(II), can decrease, or even disappear, if additional N-donor(s) is (are) situated in the molecule with chelatable position to the hydroxamate function [6–8]. This may happen, for example, with the hydroxamic derivatives of amino acids and peptides, because they are able to form very stable $\left({\text{N}}_{\text{amino}/\text{peptide}},{\text{N}}_{\text{hydroxamate}}\right)$-chelated complexes with the metal ions like Ni(II), or Cu(II) and, especially with Cu(II), very high stability polynuclear complexes (metallacrowns) involving both (O,O-hydroxamate and N,N-amine) types of chelates can also be formed [9, 10]. On the other hand, such molecules form only hydroxamate-type (O,O)-chelates with Fe(III), and both the electron-withdrawing effect of the amino group and electronic repulsion between the ${\text{NH}}_{3}^{+}$ group and the Fe(III) ion decrease the stability of the hydroxamate chelate [11]. Therefore, an aminohydroxamic acid generally forms lower stability Fe(III) complexes compared to a simple monohydroxamic acid [6]. Somewhat, more stable mono- and biscomplexes are formed with aspartic acid-*β*-hydroxamic acid and glutamic acid-*γ*-hydroxamic acid, what was assigned to the tridentate coordination of these ligands via their hydroxamate and carboxylate oxygens, but the coordination of the carboxylate cannot hinder the coordination of a third hydroxamate chelate [6]. Unique bonding mode [12] and electrochemical behavior [13] of the Fe(III)-histidinehydroxamate complexes were found by the few previous results, because the dominant role of the imidazole- and the amino-N donors and the less important part of the hydroxamate-type (O,O)-coordination were suggested. In fact, the imidazole moiety is a favored coordinating site by numerous metals [14], but this suppression of the hydroxamate-type coordination in Fe(III)complexes is quite surprising. In order to get additional information on this problem, detailed investigation of the Fe(III) complexation with histidinehydroxamic acid was carried out; furthermore, Fe(III) complexes of some other imidazole containing hydroxamic acids (the formulae of the ligands are shown in Scheme1) were studied by pH-potentiometry, UV-visible spectrophotometry, NMR, EPR spectroscopic, and ESI-MS spectrometric methods.

## 2. EXPERIMENTAL

### 2.1. Reagents and stock solutions

L-Hisha was of the highest purity commercially available (Sigma) and used without further purification. Imidazole-4-acetohydroxamic acid hydrochloride, imidazole-4-carbohydroxamic acid hydrochloride, and N-methyl-imidazole-4-carbohydroxamic acid hydrochloride were prepared as reported previously [15]. The exact concentration of the ligand stock solutions was determined by Gran's method [16].

Fe(III) stock solution was prepared by dissolving FeCl_3_ (Reanal) in dilute HCl (0.1 M). The concentration of the metal ion stock solution was determined by gravimetry, via metal oxide.

### 2.2. Potentiometric and spectroscopic studies

All the measurements were carried out at an ionic strength of 0.2 M, which was set with KCl. The temperature was always 25.0±0.1°C. Carbonate-free KOH solution (0.2 M) was used as titrant. HCl stock solution was prepared from cc. HCl. The concentrations of the KOH and HCl were determined by pH-potentiometric titrations using the Gran method [16].

A Radiometer pHM 84 with Metrohm combined electrode (type 6.0234.110) was used for pH-potentiometric measurements with Metrohm 715 Dosimat automatic burette. The electrode system was calibrated according to Irving et al. and the pH-metric readings could, therefore, be converted into hydrogen concentration [17]. The water ionization constant (pK_ W_) determined in the present work is 13.76±0.01. All the pH-potentiometric titrations were performed over the pH range of 2 to 11, or were terminated if precipitate was formed. The initial volume of the samples were 3.00 or 10.00 mL. The ligand concentration was 0.01–0.003 M in each system measured and the metal ion concentration was varied in the range of 0.0002–0.003 M, in order to get 1 : 1, 1 : 2, 1 : 3, 1 : 5, and 1 : 10 metal ion to ligand ratios in the samples. The samples were completely deoxygenated by bubbling purified argon for approximately 20 minutes before the measurements. The equilibrium calculations were performed by the PSEQUAD computer program [18]. Since the measurable hydrolysis of the Fe(III) ion starts at low pH, the hydrolytic species, with their fixed equilibrium constants, were always included in our equilibrium models when the experimental data for the Fe(III)-hydroxamate systems were fitted. The hydrolysis model and the stability of the hydroxo-complexes formed were found in the literature [19].

In addition to the generally used pH-potentiometry, the interaction between these hydroxamate-based ligands and Fe(III) was also followed by UV-visible spectrophotometry and the well-known characteristic charge transfer band was evaluated. The iron (III)-monohydroxamato complexes have spectra with $\lambda_{\max}$ at ca. 510 nm (*ε*° ∼ 1000 mol^-1^cm^-1^), the bishydroxamato ones display $\lambda_{\max}$ at ca. 470 nm (*ε*° ∼ 1800 mol^-1^cm^-1^), while $\lambda_{\max}$ of the tris-complexes is at ca. 425 nm (*ε*° ∼ 2600 mol^-1^cm^-1^) [20]. Spectrophotometry was used to clarify the complex formation below pH 2, where pH-metry could not be used. In these cases, measurements were carried out on individual samples in which the 0.2 MKCl was partially or completely replaced by HCl and the pH values, varying in the range ca. 0.7–1.4, were calculated from the HCl content.

A Perkin Elmer Lambda 25 or an Avantes fiber optic spectrophotometer equipped with a 2 mm probe head by using 20 milliseconds integration time and an average of 25 spectra were used to record the UV-visible spectra for the Fe(III)-hydroxamate complexes in the range of 300–800 nm at all metal-to-ligand ratios studied with pH-potentiometry, in the 0.0005–0.004 M metal ion concentration region.

Magnetic measurements were carried out by using the Evans method on a Bruker Advance II 300 MHz NMR spectrometer [21]. Coaxial NMR tubes were used, where the inset contained the reference D_2_O solution with 2% of *t*-BuOH, while the paramagnetic species, dissolved in D_2_O containing 2% of *t*-BuOH, was in the outher tube. The concentration of the metal ion was 0.006 M at 1 : 3 metal-to-ligand ratio. All measurements were performed without inert salt (KCl) added. pH* values, in the range of 2–10, were set up with D_2_SO_4_ or NaOD. The pH* value is a direct reading in a D_2_O solution of the “H_2_O-calibrated” pH meter. The pH* values can be converted to pH values using the following equation: pH = 0.930 pH* + 0.40 [22].

Electrospray ionization time-of-flight mass spectrometric (ESI-TOF MS) analysis was carried out for each system on a Bruker BIOTOF II ESI-TOF instrument by using 0.006 M ligand concentration at 1 : 3 metal-to-ligand ratio. The pH values were set at the Fe(III)-Im-4-Cha, -N-Me-Im-4-Cha, -Im-4-Aha and -Hisha: 3.4, 6.4, 8.2, and 7.5, respectively. The solutions were introduced directly into the ESI source by a syringe pump (Cole-Parmer Ins. Comp. type 74900.) at a flow rate of 2 *μ*L/min. The temperature of drying gas (N_2_) was 100°C. The pressure of the nebulizating gas (N_2_) was 30 psi. Voltages applied at the capillary entrance, capillary exit and the first and the second skimmers were −4500 V, 120 V, 40 V, and 30 V, respectively. The spectra were accumulated and recorded by a digitizer at a sampling rate of 2 GHz. The spectrometer was operated at unit mass resolution and was calibrated to sodium trifluoroacetate.

Anisotropic X-band EPR spectra of frozen solutions were recorded at 77 K in 100–4500 G region, using a Bruker EleXsys E500 spectrometer after the addition of 10% methanol to ensuregood glass formation. All the solutions were filtered before the measurements.

## 3. RESULTS AND DISCUSSION

### 3.1. Proton-dissociation equilibria of the ligands

The totally protonated forms of the imidazole-hydroxamic acids Im-4-Cha, N-Me-Im-4-Cha, and Im-4-Aha involve two dissociable protons each, so their general formula is H_2_L^+^. There are clear evidences in our previous paper [15] that in all of these molecules, the dissociation of the two protons occurs in almost completely separated processes and the lower pK belongs to the dissociation of imidazole-NH^+^, while the higher to the proton release from the hydroxamic moiety. Based on the exceptionally low ${\text{pK}}_{\text{a}1}$ of Im-4-Cha, the existence of hydrogen bond between the imidazole-N and hydroxamic-NH was suggested [15]. The dissociation constants taken from 14 are shown in Table 1.

**Figure 1:**
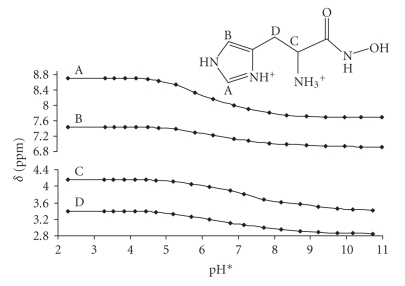
pH* dependence of the ^1^H NMR chemical shifts recorded for Hisha, ${\text{c}}_{\text{ligand}}$ = 3 ⋅ 10^-3^ M.

In contrast to the situation of the above three imidazole derivatives, the dissociation of the protons from the completely protonated Hisha (H_3_L^2+^) occurs in overlapping processes, what is well demonstrated by the pH-dependence of the chemical shifts of the nonlabile protons (Figure 1). As a consequence, pK values shown in Table 1 are macroconstants, characterize the proton releasing at molecular level, but cannot be designated to the individual moieties. However, it is clearly shown by Figure 1 that out of the three groups, the imidazole-NH^+^ is the most acidic.

### 3.2. Fe(III) complexes of the ligands

**Complexes with Im-4-Cha, N-Me-Im-4-Cha, and Im-4-Aha**

**Scheme 2:**
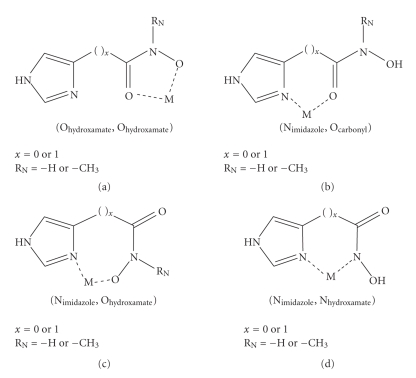


In these molecules, the donor atoms are the imidazole-nitrogen, the hydroxamate oxygens, and (except N- Me-Im-4-Cha, where this donor is not available) the hydroxamate nitrogen. The most possible chelation modes between these ligands and a metal ion are summarized in Scheme 2.

It is beyond doubt that, most of all, the coordination mode (a) can be expected with Fe(III). Conditional stability of (b) is much lower than (a) even with less hard metal ions than Fe(III) [15], so its formation is not probable here. The mode (d) is excluded with N -Me-Im-4-Cha, while the mode (c) would result in the formation of a low-stability seven-membered chelate with Im-4-Aha. It is easy to understand that (b) and (c) cannot be formed at the same time with the same molecule, while polynuclear complexes are expected via parallel formation of (a) and (d). Following this theoretical speculation, the obtained results are summarized below.

**Figure 2:**
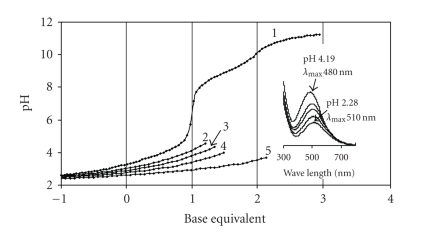
pH-potentiometric titration curves registered for Im-4-Cha (**1**) and Fe(III)–Im-4-Cha systems at metal-to-ligand ratio 1 : 5 (**2**), 1 : 3 (**3**), 1 : 2 (**4**) and 1 : 1 (**5**); Inset: selected UV-VIS spectra registered for the Fe(III)–Im-4-Cha as a function of pH at 1 to 5 metal-to-ligand ratio c_ligand_ = 3 ⋅ 10^-3^ M.

With **Im-4-Cha** precipitation occurred at pH 3-4 in presence of Fe(III) (this is the reason why the titration curves are interrupted in Figure 2), therefore only a few species could be detected before precipitation.

The equilibrium model yielding the best fit of the titration curves together with the overall stability constants is shown in Table 2.

**Scheme 3:**
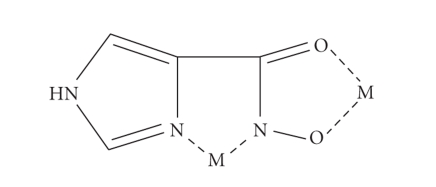


A comparison of the corresponding constants of the complexes of Im-4-Cha with those of a simple monohydroxamic acid, such as acetohydroxamic acid, CH_3_CONHOH (log *β*_FeL_ = 11.09, log $\beta_{{\text{FeL}}_{2}}$ = 20.69 [20]), supports the predominance of the hydroxamate-type coordination mode in the water-soluble Fe(III)-Im-4-Cha complexes. The same conclusion can be drawn from UV-visible spectra detected at different ratios (some of them are presented in the inset in Figure 2). The charge transfer band indicates appearance of (O,O)-hydroxamate-Fe(III) interaction above ca pH 1. The formation of the monochelated complex ($\lambda_{\max}$ = 510 nm) is completed by pH ca. 2.5 (this depends somewhat on the metal-to-ligand ratio) and bischelated complex(es) ($\lambda_{\max}$ = 470 nm) could also be detected before the precipitation. Due to the very low basicity of the imidazole-N, formation of protonated complexes is not really favored with this ligand and, as a consequence, [FeLH]^3+^ is the only one, what appears in low concentration (max. 10%) at very low pH. The unexpectedly poor solubility of the complexes formed above pH 3-4 indicates involvement of the imidazole in the interaction (Generally, even the neutral, tris-chelated Fe(III)-complexes of small monohydroxamic acid molecules have good water solubility.) (one possibility for this is presented in Scheme 3, demonstrating formation of polynuclear complexes, which might have low water solubility). Unfortunately, we still have not been successful to get any direct information on the stoichiometry and bonding mode of the solid-state complexes formed.

**Figure 3:**
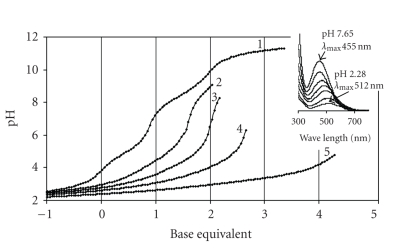
pH-potentiometric titration curves registered for N-Me-Im-4-Cha (**1**) and Fe(III)-N-Me-Im-4-Cha systems at metal-to-ligand ratio 1 : 5 (**2**), 1 : 3 (**3**), 1 : 2 (**4**) and 1 : 1 (**5**). Inset: selected UV-VIS spectra registered for the Fe(III)-Im-4-Cha as a function of pH at 1 to 5 metal-to-ligand ratio c_ligand_ = 3 ⋅ 10^-3^ M.

Much more soluble complexes are formed with N-Me-Im-4-Cha as it is demonstrated by Figure 3, what, moreover, clearly shows metal-induced deprotonation of both the hydroxamic and imidazole-NH^+^ protons.

Fitting the experimental data resulted in the model in Table 2. This model involves mono-, bis-, and tris-complexes. Out of them, the latter two were also supported by ESI-MS at 1 : 3 ratio, at pH = 8.2 (characteristic m/z values for the sodium salts are 359, 499). However, if the stability constants shown for the complexes in Table 2 are analyzed, one can find them somewhat higher than expected for simple hydroxamate-type complexes (Scheme 2(a)). Just for comparison, for N-Me-acetohydroxamic acid the pK = 8.70 and the overall stability constants are log *β*_FeL_ = 11.85, log $\beta_{{\text{FeL}}_{2}}$ = 21.58, while log $\beta_{{\text{FeL}}_{3}}$ = 29.36 [20]. Also the minimum $\lambda_{\max}$ value, what would be expected in the range of 425–430 nm for a tris-hydroxamato complex, here is ca. 455 nm (see inset in Figure 3). The most possible explanation for all the above findings is that not only one type of bonding modes belongs to the complexes with the same stoichiometry, but different bonding isomers exist. For example, [FeL]^2+ ^ may involve either hydroxamate (O,O)-type chelate (Scheme 2(a)) or $\left({\text{N}}_{\text{imidazole}},{\text{O}}_{\text{hydroxamate}}\right)$-type one (Scheme 2(c)). This situation was previously suggested between N-Me-hydroxamic acids and several metal ions [15]. Existence of mixed type chelated complexes is suggested in the bis- and tris-complexes.

**Figure 4:**
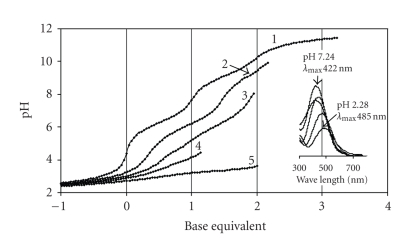
pH-potentiometric titration curves registered for Im-4-Aha (**1**) and Fe(III)-Im-4-Aha systems at metal-to-ligand ratio 1 : 5 (**2**), 1 : 3 (**3**), 1 : 2 (**4**) and 1 : 1 (**5**). Inset: selected UV-VIS spectra registered for the Fe(III)-Im-4-Cha as a function of pH at 1 to 5 metal-to-ligand ratio c_ligand_ = 3 ⋅ 10^-3^ M.

 Im-4-Aha involves the imidazole moiety in *β*-position to the hydroxamic one. The pH-metric titration curves for the Fe(III)-Im-4-Aha samples are presented together with that of the free ligand in Figure 4.

**Figure 5:**
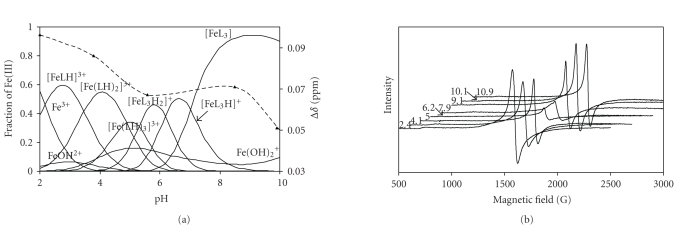
Representative concentration distribution curves calculated for Fe(III)-Im-4-Aha system at 1 to 3 metal-to-ligand ratio, showing also pH dependence of the differences between the ^1^H NMR chemical shifts (Δ*δ* values) of *t*-BuOH in dia- and paramagnetic surroundings at the same metal-to-ligand ratio (dashed line) (a), and EPR spectra registered for Fe(III)-Im-4-Aha system at 1 to 3 metal-to-ligand ratio at different pH values (b), T = 77 K; c_ligand_ = 3 ⋅ 10^-3^ M.

Figure 4 shows that if there is enough ligand excess, there is no precipitation up to pH ca. 8–10 (curves 2 and 3) in the samples, but if not, hydroxo species precipitate at pH ca. 4, or somewhat below. It is also clear that the imidazole-NH^+^, which dissociates in the free ligand in the pH-range 5–7 (Figure 4, curve 1), is released almost in the same range from the Fe(III)-complexes (curves 2 and 3). It means that this proton is not displaced by the metal ion, consequently, the imidazole-N of Im-4-Aha does not coordinate to the Fe(III) ion. In accordance with these findings, the equilibrium model involves large amount of protonated complexes, in which the imidazole-N still contains the proton (see Table 2) and only hydroxamate type coordination of the ligands occurs. The UV-visible results agree completely with this suggestion (see Inset in Figure 4), but there is some doubt if we look at the EPR and magnetic moment results. To demonstrate this, together with the concentration distribution curves at 1 : 3 metal-to-ligand ratio, differences between the chemical shifts of *t*-BuOH in dia- and paramagnetic surroundings as a function of pH are shown in Figure 5(a). (Mass magnetic susceptibilities calculated by the Evans method from these NMR data [21] show completely the same profile, therefore are not presented here.) Figure 5(b) shows the EPR spectra recorded at various pH.

Figures 5(a) and  5(b) show that there is some decrease both in the NMR Δ*δ* values and in the intensity of the EPR signal within the pH range ca. 5–7, where first of all the [FeL_3_H_2_]^2+^ and [FeL_3_H]^+^ complexes dominate. Possible explanation of these results is that some intermolecular interaction (H-bond) between the already nonprotonated and the still-protonated imidazole rings exists, resulting in some coupling between the paramagnetic metal centers.

**Complexes with Hisha**

**Figure 6:**
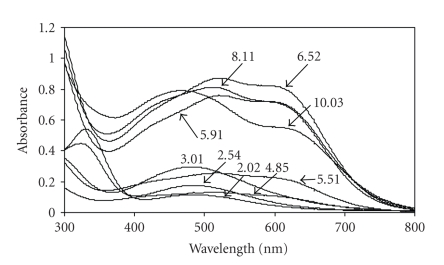
Selected UV-VIS spectra registered in the Fe(III)-Hisha system as a function of pH at 1 to 10 metal-to-ligand ratio; c_ligand_ = 5*⋅*10^−3^ M.

As it is shown in Scheme 1, in this ligand, the R_N_ substituent also involves an amino moiety in addition to the imidazole function. A further difference is that the imidazole-N is situated in *γ*-position to the hydroxamic moiety (what is *α* in the Im-4-Cha or N-Me-Im-4-Cha and *β* in the Im-4-Aha). Due to these structural differences, the Fe(III)-binding behavior of Hisha is also different from that of the above detailed imidazole-hydroxamic acids. To demonstrate this difference, some of the registered UV-visible spectra as a function of pH are shown in Figure 6.

As Figure 6 shows, there is some complex formation between Fe(III) and Hisha already at pH 2, and the *λ*_max_ at ca. 500 nm suggests the presence of an Fe(III)-monohydroxamato (Scheme 2(a)) species, but some bishydroxamato complex can also be present. At this pH, however, and also in all the spectra registered below ca. pH 2.5–3, the presence of some Fe(III)-chloro complexes can be detected too (characteristic *λ*_max_ ca. 340 nm) [23]. With increasing the pH from 2 to ca. 4, *λ*_max_ shifts from 500 nm to 470 nm and also the molar absorptivity increases, which suggests the dominance of the bishydroxamato complex at the latter pH. On increasing the pH further, dramatic change in the spectrum occurs (This change in the spectra was practically the same at all the ratios studied, except 1 : 1, where precipitate was formed above pH 3.5.). First, a significant decrease in the intensity can be observed, then the intensity of a broadband (superposition of two bands with *λ*_max_ values 620 and ca. 500 nm) increases up to pH ca. 7. Above this pH, the peak at 620 nm decreases intensively, while the other shifts to the lower wavelengths.

All the above findings (together with the only previous results [12]) have been taken into account when the pH-potentiometric titration curves were fitted. Calculations have been done with numerous reasonable models, including tris- and dinuclear complexes, but unambiguously, the best fitting was obtained with the model shown in Table 3 (e.g., if tris-complexes were also involved, the fitting was 1.03 ⋅ 10^-2^ and the standard deviations were unacceptably high). As a consequence, the equilibrium model shown in Table 3 differs from the previously published one [12]. Namely, tris-hydroxamato complexes were not found to form in measurable concentration under our conditions even at 1 : 10 metal-to-ligand ratio (although, the shoulder at ca. 430 nm being on the spectra from pH 5.91 in Figure 6 might indicate the presence of small amount of tris-complexes) (maximum 1 : 20 ratio was used in [12], where measurable formation of tris-complexes was found).

**Figure 7:**
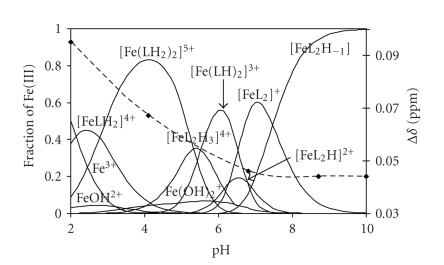
Representative concentration distribution curves calculated for Fe(III)-Hisha system at 1 to 3 metal-to-ligand ratio, on the secondary axis, pH dependence of the differences between the ^1^H NMR chemical shifts (Δ*δ* values) of *t*-BuOH in dia- and paramagnetic surroundings for Fe(III)-Hisha (dashed line) at 1 to 3 metal-to-ligand ratio, c_ligand_ = 3 ⋅ 10^-3^ M.

By using the accepted model and stability constants, concentration distribution curves were calculated and are presented in Figure 7.

According to our model (see Table 3 and Figure 7), the complex formation starts with the appearance of [FeLH_2_]^4+^ in measurable concentration. Most probably, the hydroxamate oxygens coordinate to the metal ion, while the amino group and the imidazole-N are still protonated in this species. The next complex [Fe(LH_2_)_2_]^5+^ is suggested to involve the two ligands in the same (hydroxamate-type) coordination mode. The stepwise deprotonation of this latter complex starts above pH ca. 3.5–4 and, parallel, the Δ*δ* values (namely the magnetic susceptibility) are decreasing. If the Δ*δ* values, obtained here, are compared to those measured under the same condition for the Fe(III)-Im-4-Aha (Figure 5(a)) and Fe(III)-acetohydroxamic (Aha) systems, (Δ*δ* values registered in this work for the Fe(III)-acetohydroxamic acid sample at 1 : 3 ratio are 0.110±0.005 ppm in the pH-range 2–9, and intensively starts to decrease above pH 9) one can find that much lower values belong to the Fe(III)-Hisha system than to the Fe(III)-Im-4-Aha and especially to the Fe(III)-Aha. The UV-visible spectra, as it was detailed above (Figure 6), also show significant changes. A comparison of Figures 6 and  7 shows that the decrease of the absorbance starts, where the concentration of [Fe(LH_2_)_2_]^5+^ starts to decrease (parallel, the concentration of [Fe(L_2_H_3_)]^4+^ then [Fe(LH)_2_]^3+^ increases), where, based on the acidities, first of all the imidazole-NH^+^ moieties are assumed to deprotonate. However, dramatic increase of the absorbance occurs, where the coordinated ligands start to release their last dissociable protons from the -NH_3_^+^ groups.

The above findings, together with the EPR results, what showed that exclusively EPR silent species exist in the Fe(III)-Hisha system at 1 : 3 ratio above pH 4, indicate metal-metal coupling in this pH-region. Since stepwise deprotonation of [Fe(LH_2_)_2_]^5+^ is accompanied by the above detailed changes, this suggests the involvement of the nonprotonated side-chain donors (imidazole-N and, at higher pH, perhaps the amino-N) in the coordination. Either coordinative or noncovalent (H-bond, stacking) interaction of imidazole-N can be assumed, which, at least in some extent, should be intermolecular (e.g., H-bond between the already nonprotonated and still-protonated imidazoles, or between the imidazole-N and ammonium-${\text{NH}}_{3}^{+}$ protons) resulting in some metal-metal coupling. Most probably, these interactions are able to hinder the acceptance of the third hydroxamate chelate by the metal ion at lower pH (below pH ca. 7) in high extent. At higher pH, the formation of mixed hydroxo species seems the most possible. The existence of this latter species is also supported by the ESI- MS results. Two different iron-ligand-containing species can be detected in the positive region in the spectrum at pH 7.5 (m/z values 394 and 435). The first can be assigned unambiguously to [FeL_2_]^+^, while the second perhaps to [FeL(LH)(OH)]Na^+^.

## 4. CONCLUDING REMARKS

All the above results summarized for the systems containing Fe(III) ion and Im-4-Cha, N-Me-Im-4-Cha, Im-4-Aha, or Hisha demonstrate that the imidazole moiety is able to play an important role very often in the interaction with Fe(III), even if this metal ion prefers the hydroxamate chelates very much. Moreover, each of the studied ligands forms complexes with different bonding modes.

If the imidazole and hydroxamic moieties are in *α*-position to each other (Im-4-Cha, or *N*-Me-Im-4-Cha), coordinative bonding of the imidazole seems possible. This results in the formation of various poorly soluble polynuclear species with Im-4-Cha, while the appearance of the interesting N_imidazole_, O_hydroxamate_ chelate seems probable with N-Me-Im-4-Cha.

In Im-4-Aha, the imidazole is in *β*-position to the hydroxamic moiety and seems to organize the intermediate-protonated molecules in the solution via noncovalent (first of all H-bonding) interaction. This interaction otherwise cannot hinder the coordination of up to three hydroxamate chelates to an Fe(III) ion, if there is enough ligand excess in the solution.

The situation is quite different with Hisha, in which the position of the imidazole-N is *γ* to the hydroxamic moiety and there is also an amino group in the molecule. The formation of this hydroxamate-type complex was not found in measurable concentration under the investigated conditions (up to 1 : 10 metal-to-ligand ratio). This suggests rather significant role of the imidazole (perhaps together with ammonium) in the interaction.

## Figures and Tables

**Table 1:** Dissociation constants (pK)^(a)^ for the investigated imidazole-containing ligands, *t* = 25°C, *I* = 0.2 M (KCl).

Ligands	Im-4-Cha^(b)^	N-Me-Im-4-Cha^(b)^	Im-4-Aha^(b)^	Hisha^(c)^
pK_1_	3.76	5.06	6.17	5.38(4)
pK_2_	8.82	8.57	9.05	7.11(3)
pK_3_	—	—	—	9.06(2)

^(a)^Standard deviations are shown in parenthesis. ^(b)^See [15]. ^(c)^The values determined for Hisha in the present work are in good agreement with the previously published data in [12].

**Table 2:** Overall stability constants for the complexes formed in the Fe(III)-Im-4-Cha, Fe(III)-N-Me-Im-4-Cha and Fe(III)-Im-4-Aha systems* *I* = 0.2 M (KCl), *t* = 25°C.

Ligands	Im-4-Cha	N-Me-Im-4-Cha	Im-4-Aha
[FeLH]^3+^	12.1(2)	13.95(3)	15.62(2)
[FeL]^2+^	10.46(1)	11.92(4)	—
[FeL_2_H_2_]^3+^	—	—	30.11(2)
[FeL_2_]^+^	18.65(4)	23.04(4)	—
[FeL_2_H_−1_]	—	18.8(1)	—
[FeL_3_H_3_]^3+^	—	—	43.47(6)
[FeL_3_H_2_]^2+^	—	—	38.17(4)
[FeL_3_H]^+^	—	—	31.99(5)
FeL_3_	—	31.3(2)	24.97(5)

*Standard deviations are shown in parenthesis.

**Table 3:** Overall stability constants for the complexes formed in the Fe(III)-Hisha system*; *I* = 0.2 M (KCl), *t* = 25°C.

Species	log *β*
[FeLH_2_]^4+^	21.98(6)
[Fe(LH_2_)_2_]^5+^	43.45(3)
[FeL_2_H_3_]^4+^	38.11(7)
[Fe(LH_2_)]^3+^	32.59(5)
[FeL_2_H]^2+^	25.81(9)
[FeL_2_]^+^	19.53(5)
[FeL_2_H_−1_]	12.05(5)
Number of points fitted	292
Fitting (cm^3^)	8.4*⋅*10^−3^

*Standard deviations are shown in parenthesis.
